# Multiple roles for Na_V_1.9 in the activation of visceral afferents by noxious inflammatory, mechanical, and human disease–derived stimuli

**DOI:** 10.1016/j.pain.2014.06.015

**Published:** 2014-10

**Authors:** James R.F. Hockley, George Boundouki, Vincent Cibert-Goton, Cian McGuire, Ping K. Yip, Christopher Chan, Michael Tranter, John N. Wood, Mohammed A. Nassar, L. Ashley Blackshaw, Qasim Aziz, Gregory J. Michael, Mark D. Baker, Wendy J. Winchester, Charles H. Knowles, David C. Bulmer

**Affiliations:** a Wingate Institute of Neurogastroenterology, Blizard Institute, Barts and the London School of Medicine and Dentistry, Queen Mary University of London, London E1 2AJ, UK; b National Centre for Bowel Research and Surgical Innovation, Blizard Institute, Barts and the London School of Medicine and Dentistry, Queen Mary University of London, London E1 2AT, UK; c Centre for Neuroscience and Trauma, Blizard Institute, Barts and the London School of Medicine and Dentistry, Queen Mary University of London, London E1 2AT, UK; d Molecular Nociception Group, Wolfson Institute for Biomedical Research, University College London, London WC1E 6BT, UK; e Department of Biomedical Science, The University of Sheffield, Sheffield S10 2TN, UK; f Neusentis (Pfizer Ltd), The Portway Building, Granta Science Park, Cambridge CB21 6GS, UK

**Keywords:** Distal colon, Inflammatory bowel disease, Na_V_1.9, Nociceptor sensitivity, Noxious distension, Supernatants, Visceral hypersensitivity, Visceral pain, Voltage-gated sodium channel

## Abstract

Chronic visceral pain affects millions of individuals worldwide and remains poorly understood, with current therapeutic options constrained by gastrointestinal adverse effects. Visceral pain is strongly associated with inflammation and distension of the gut. Here we report that the voltage-gated sodium channel subtype Na_V_1.9 is expressed in half of gut-projecting rodent dorsal root ganglia sensory neurons. We show that Na_V_1.9 is required for normal mechanosensation, for direct excitation and for sensitization of mouse colonic afferents by mediators from inflammatory bowel disease tissues, and by noxious inflammatory mediators individually. Excitatory responses to ATP or PGE_2_ were substantially reduced in Na_V_1.9^−/−^ mice. Deletion of Na_V_1.9 substantially attenuates excitation and subsequent mechanical hypersensitivity after application of inflammatory soup (IS) (bradykinin, ATP, histamine, PGE_2_, and 5HT) to visceral nociceptors located in the serosa and mesentery. Responses to mechanical stimulation of mesenteric afferents were also reduced by loss of Na_V_1.9, and there was a rightward shift in stimulus–response function to ramp colonic distension. By contrast, responses to rapid, high-intensity phasic distension of the colon are initially unaffected; however, run-down of responses to repeat phasic distension were exacerbated in Na_V_1.9^−/−^ afferents. Finally colonic afferent activation by supernatants derived from inflamed human tissue was greatly reduced in Na_V_1.9^−/−^ mice. These results demonstrate that Na_V_1.9 is required for persistence of responses to intense mechanical stimulation, contributes to inflammatory mechanical hypersensitivity, and is essential for activation by noxious inflammatory mediators, including those from diseased human bowel. These observations indicate that Na_V_1.9 represents a high-value target for development of visceral analgesics.

## Summary

Na_V_1.9 regulates normal colonic afferent mechanosensation and is required for hypersensitivity to noxious inflammatory mediators and those derived from inflammatory bowel disease tissues.

## 1 Introduction

Chronic dysregulation of visceral sensation can lead to abdominal pain, which is clinically challenging to treat. A hallmark of chronic visceral pain is heightened sensitivity to gut motility and distension of visceral organs, typically in response to past or ongoing inflammation [26]. In some patient groups, therapeutic inhibition of mechanosensitivity in visceral afferents has proven effective in treating pain [51], [52]. However, opportunities for improved intervention will likely come from targeting mechanisms of activation common to both inflammatory and mechanical stimulation of the viscera.

The activity of voltage gated sodium channels (Na_V_) underpins electrogenesis in excitable cells. The 9 genes (*SCN1A–SCN5A* and *SCN8A–SCN11A*) encoding the Na_V_1.1 to Na_V_1.9 α-subunits exhibit tissue specific expression and describe proteins with varying biophysical characteristics. Of those expressed in peripheral neurons, 3 channels (Na_V_1.7, Na_V_1.8, and Na_V_1.9) are strongly associated with pain behaviours in both rodents and humans. Specifically, Na_V_1.9 mediates a slowly inactivating persistent sodium current that is activated at voltages close to the resting membrane potential of sensory neurones. As a consequence of these biophysical properties, Na_V_1.9 is proposed to contribute to the resting membrane potential and regulate nerve terminal excitability [3]. More recently, we, as well as others, have shown that the Na_V_1.9 sodium current may be enhanced by the algogenic mediator, ATP, and the inflammatory mediator PGE_2_, acting through G protein–coupled pathways, suggesting that Na_V_1.9 may also play an important role in the activation of sensory nerves by inflammatory mediators [2], [30], [44]. This hypothesis is supported for somatic pain by behavioural studies demonstrating reduced hypersensitivity to inflammatory stimuli in rodents where Na_V_1.9 has been deleted or knocked down [1], [28], [39]. However, evidence for a role of Na_V_1.9 in visceral pain processing is controversial, with behavioural studies in Na_V_1.9 knockout mice reporting either reduced or enhanced pain behaviours to noxious stimuli [27], [31], [41]. Similarly the extent to which Na_V_1.9 is expressed within visceral afferents is far from clear [4], [24]. As a consequence, the contribution of Na_V_1.9 to visceral afferent sensitivity remains to be defined.

We hypothesized that the persistent sodium current mediated by Na_V_1.9 plays a significant role in the polymodal sensitivity of visceral afferents to inflammatory mediators and the subsequent development of hypersensitivity to mechanical stimuli. We therefore investigated effects of Na_V_1.9 gene deletion on the response of visceral afferents to inflammatory and mechanical stimuli, and Na_V_1.9 expression in colon-projecting sensory neurons.

## 2 Materials and methods

All experimental studies were performed in accordance with the UK Animal (Scientific Procedures) Act of 1986. Human tissue was collected and used with approval of the East London and the City HA Local Research Ethics Committee (NREC 10/H0703/71).

### 2.1 In vitro mouse colonic splanchnic afferent preparations

Na_V_1.9^−/−^ mice were rederived from Na_V_1.9^+/−^ breeding pairs and originally generated by homologous recombination on a C57/BL6 background, as described previously [36]. Adult Na_V_1.9^+/+^ or Na_V_1.9^−/−^ mice of either sex were euthanized by rising concentration of CO_2_, and the distal colon with associated lumbar splanchnic nerves removed. For whole-nerve experiments, tissues were cannulated, luminally perfused (100 μL/min), and serosally superfused (7 mL/min; 32–34°C) with carbogenated Krebs buffer (in mM: 124 NaCl, 4.8 KCl, 1.3 NaH_2_PO_4_, 2.5 CaCl_2_, 1.2 MgSO_4_·7H_2_O, 11.1 glucose, and 25 NaHCO_3_) supplemented with nifedipine (10 μM) to block smooth muscle contraction, and indomethacin (3 μM) to block endogenous prostanoid production. Atropine (10 μM), the muscarinic acetylcholine receptor antagonist, was also added to the Krebs buffer to further prevent smooth muscle contractions. For single-fibre recordings, tissues were perfused with supplemented Krebs as described above; however, the colon was opened along the antimesenteric border and pinned with the flat mucosal side up. This preparation has been described in detail previously [6], [8], [19].

### 2.2 Electrophysiological recordings and characterization of colonic splanchnic afferent properties

Borosilicate glass suction electrodes were used to record multiunit activity from whole lumbar splanchnic nerves (rostral to the inferior mesenteric ganglia) or single-unit activity from fibres teased from lumbar splanchnic nerves. Signals were amplified, band pass filtered (gain 5 K; 100–1300 Hz; Neurolog, Digitimer Ltd, UK), and digitally filtered for 50 Hz noise (Humbug, Quest Scientific, Canada). Raw traces were digitized at 20 kHz (micro1401; Cambridge Electronic Design, UK), and action potential firing counts were determined using a threshold of twice the background noise (typically 100 μV). All signals were displayed on a PC using Spike 2 software. In flat sheet preparations, distinct receptive fields were identified and characterized according to previously published classifications by graded stimulus–response to punctate von Frey hair (vFh) probing (0.07 g, 0.16 g, 0.4 g, 1 g, and 2 g; each applied 3 times for a period of 3 s), circumferential stretch (0 g, 5 g, and 10 g; each weight applied for 1 min, with an interval of 1 min between applications), and mucosal stroking with light vFh (0.16 g; applied 10 times) [6], [19], [21]. A cantilever system was used to apply stretch via a thread attached with a purpose-made claw to the tissue adjacent to the receptive field. Addition of weights to the end of the cantilever system initiated colonic stretch. Four distinct classes of afferent fibre were identified on the basis of these responses: muscular (those responding to low-intensity circumferential stretch [⩽5 g], but not fine mucosal stroking); mucosal (those responding to light vFh stroking), mesenteric (those responding to focal compression of the mesentery), and serosal (those responding to focal compression of the colon wall, but not low-intensity stroke or low-intensity circular stretch). In addition, conduction velocity was calculated for serosal and mesenteric units by dividing evoked action potentials latency elicited after electrical stimulation (0.5 Hz, 15 V, 1 ms) of the receptive field with concentric stimulating electrodes by the distance from the recording electrode to receptive field.

### 2.3 Retrograde labelling of colonic sensory neurones

Fast Blue (FB; 2% in saline, Polysciences Gmbh, Germany) was injected into the wall of the distal colon of Na_V_1.9^+/+^ and Na_V_1.9^−/−^ mice and male Sprague Dawley rats (150–250 g). Briefly, animals were anaesthetized with isoflurane then an approximate 1.5 cm laparotomy performed. Five injections of 0.2 μL FB per site, at a rate of 0.4 μL/min via a microinfusion pump, were made into the wall of the distal colon using a fine-pulled glass needle. The muscle layer was sutured and the skin secured with Michel clips. Postoperative analgesia (buprenorphine 0.05–0.1 mg/kg daily) and care (monitoring body weight and soft diet) was provided.

After 3 days for mice and 7 days for rats, animals were euthanized with sodium pentobarbital (200 mg/kg i.p.) and transcardially perfused with saline (0.9%) followed by paraformaldehyde (4% in 0.1 M phosphate buffer; pH 7.4). Dorsal root ganglia (DRG; Na_V_1.9^+/+^ and Na_V_1.9^−/−^ mice, T13–L1; rats, L2) were removed and postfixed in 4% paraformaldehyde for 2 h and cryoprotected in 30% sucrose (w/v phosphate-buffered saline). Cryostat sections (10 μm) were collected sequentially across 10 slides per DRG.

### 2.4 Immunohistochemistry

Sections were blocked in antibody diluent (10% horse serum and 0.2% (v/v) Triton X-100 in 0.1 M phosphate-buffered saline) for 1 h, followed by overnight incubation with primary antibodies (rabbit anti-Na_V_1.9 antibody [1:1000; Alomone, Israel] and goat anti-CGRP antibody [1:2000; Abcam, UK]) and a 4 h incubation with fluorophore conjugated secondary antibodies (donkey anti-rabbit IgG-Alexafluor-488 and donkey anti-goat IgG-Alexafluor-568 [1:1000; Invitrogen, UK]) and/or isolectin B4 [IB4] from *Griffonia simplicifolia*–Alexafluor-647 (2.5 μg/mL; Invitrogen, UK). No labelling was observed in control experiments where the primary antibody was excluded or in the presence of a competing blocking peptide.

### 2.5 In situ hybridization

Oligonucleotide probes complementary to bases 968 to 1001 (probe 1) and 2641 to 2674 (probe 2) of the mouse *SCN11A* sequence, accession number NM 011887.3, were synthesized (Sigma, UK), end labelled with ^35^S, and hybridized to DRG sections using previously described protocols and visualized by silver grain development in radiographic emulsion [32]. No specific labelling was observed in DRG sections of Na_V_1.9^−/−^ mice hybridized with *SCN11A* probes; reactions where an excess of unlabelled probe was used resulted in only background signal.

### 2.6 Imaging and quantitation

Sections were imaged and the relative intensities of reaction products after immunostaining for Na_V_1.9 and other markers and *SCN11A* mRNA in situ hybridization were determined for all DRG cells with visible nuclei (ImageJ 1.45S analysis software, NIH, USA). The mean background absorbance was subtracted to control for variability in illumination. Percentage relative intensities of background-subtracted cell absorbance were determined by comparison with least intensely (0%) and most intensely (100%) labelled profiles. For in situ hybridization, relative intensity of DRG cells was measured in Na_V_1.9^−/−^ sections to set the threshold for positive labelling in wild-type mice. Cells with intensity values greater than the mean intensity of the 10 cells with highest background intensity values in Na_V_1.9^−/−^ sections plus 2 times its SD were considered positively labelled. For Na_V_1.9, CGRP and IB4-like immunostaining, intensity of staining was scored on a scale of 0 to 5 by 2 independent observers, with 0 representing negative and 5 strongly positive. The mean absorbance of these cells taken from ImageJ analysis correlated with staining scores (eg, Pearson *r* = 0.87; *P* < .0001; *n* = 149) and a threshold for positive staining was determined as performed by Fang et al. [13] (eg, mean absorbance for Na_V_1.9 ⩾ 32%).

### 2.7 Generation of human tissue supernatants

Resected human colon was obtained after full written consent was obtained from patients undergoing elective surgery at Barts Health NHS Trust, London, after approval by the local research ethics committee (NREC 10/H0703/71). Patient details are outlined in Table 1. Control supernatants were generated using macroscopically normal colon (>10 cm from tumour margin) obtained from patients (male/female ratio, 2/1; mean age, 61 years) undergoing colectomy as part of their normal surgical treatment for bowel cancer. Disease tissue supernatants were derived from chronically inflamed colon or intestine obtained from patients undergoing operations as part of their standard surgical treatment for Crohn disease (CD) (male/female ratio, 7/3; mean age, 30 years) or ulcerative colitis (UC) (male, 4; mean age, 22 years). All patients had active disease which was unresponsive to medical treatment. Tissue samples were incubated in fresh carbogenated Krebs buffer at 37°C for 25 min at a fixed volume of 2.5 mL/g of tissue. After incubation, the tissue was removed and the buffer centrifuged at 2000 × *g* for 10 min. The remaining supernatant was formed into aliquots and stored at −80°C until use.

**Table 1:** Patient details of human tissue used in the present studies.^a^

Patient no.	Disease	Operation	Age	Sex	Cytokine analysis	Whole nerve	Single fibre
1	Cancer	Laparoscopic anterior resection	50	F	Y	Y	
2	Cancer	Right hemicolectomy	76	M	Y	Y	
3	Cancer	Subtotal colectomy	57	M	Y	Y	
		*Mean age/M:F ratio*	*61*	*2:1*			
5	CD	Laparotomy and proceed	21	F	Y	Y	Y
6	CD	Redo right hemicolectomy	30	F	Y	Y	Y
7	CD	Right hemicolectomy	21	M	Y	Y	
8	CD	Completion colectomy	25	M		Y	
9	CD	Subtotal colectomy	30	M			Y
10	CD	Small bowel resection	24	F			Y
11	CD	Small bowel resection	42	M			Y
12	CD	Colectomy and end ileostomy	28	M			Y
13	CD	Extended right hemicolectomy	16	M			Y
14	CD	Subtotal colectomy	64	M			Y
		*Mean age/M:F ratio*	*30*	*2.3:1*			
15	UC	Subtotal colectomy	21	M	Y	Y	
16	UC	Restorative proctocolectomy	20	M	Y	Y	
17	UC	Subtotal colectomy	30	M	Y	Y	
18	UC	Restorative proctocolectomy	18	M	Y	Y	
		*Mean age/M:F ratio*	*22*	*4:0*			

CD, Crohn disease; UC, ulcerative colitis.

a Supernatants generated from these tissues have been used for inflammatory cytokine quantification (cytokine analysis), 20 mL bath superfusion of whole-nerve recordings from the lumbar splanchnic nerve (whole nerve), or discrete ring application to characterized receptive fields in a flat sheet preparation and single-fibre recording from the lumbar splanchnic nerve (single fibre).

### 2.8 Inflammatory cytokine quantification

Quantitative analysis of protein cytokine levels was performed on supernatant samples using established capture sandwich immunoassay and magnetic microsphere methodology [34]. Samples were prepared as per manufacturer’s instructions (Invitrogen, UK) and analysed with the Luminex MAGPIX detection system (Luminex, TX, USA) for IL-1β, IL-6, GM-CSF, TNF-α, and IL-8.

### 2.9 Electrophysiology protocols

In multiunit experiments, drugs were applied after a stabilizing period of 30 min by bath superfusion of a 20 mL volume. Where repeat concentrations of drugs were given, a minimum 60 min interval was maintained. Increasing concentrations of ATP (0.1, 1.0, and 3.0 mM) or PGE_2_ (3 μM) were applied in separate colonic preparations. Supernatants derived from CD, UC, and control tissues were applied by 20 mL bath superfusion in separate colonic preparations. Ramp distensions were performed by blocking luminal perfusion out-flow of the cannulated colon producing noxious pressures known to both evoke pain behaviours in vivo in mice and robustly activate all known afferent mechanoreceptors (0–80 mm Hg; [21], [35]). In later studies, ramp distensions from 0 to 145 mm Hg (∼4–5 min) were conducted to provide a supraphysiological pressure. Response to 0 to 80 mm Hg ramp distension was also investigated after pretreatment (20 min) with and in the presence of intraluminally perfused IS. In some studies, ramp distensions were performed once firing rates had returned to baseline activity after drug application. Sets of 6 rapid phasic ramp distensions (0–80 mm Hg, 60 s at 9 min intervals) were implemented in separate experiments. Single-fibre studies were only performed on muscular, mesenteric, or serosal units as a result of the sparse nature of mucosal splanchnic afferents. In all 3 types of units, mechanosensitivity was examined in response to graded vFh probing (0.07 g, 0.16 g, 0.4 g, 1 g, and 2 g) and circumferential stretch (0 g, 5 g, and 10 g). In mesenteric and serosal units, the effect of applying either an IS (consisting of 1 μM bradykinin, 1 mM ATP, 10 μM histamine, 10 μM PGE_2_, and 10 μM 5HT; 2 min) or CD inflammatory supernatant (5 min) to receptive fields isolated by a metal ring was also examined on ongoing nerve discharge. Additionally, changes in the response to vFh probing with a 2 g hair were examined before and after application of IS or supernatant.

### 2.10 Data analysis

In multiunit experiments, peak changes and time profiles of electrophysiological nerve activity were determined by subtracting baseline firing (5 min before drug application) from increases in nerve activity after drug/supernatant superfusion or ramp distension. Changes in nerve activity were compared between Na_V_1.9^+/+^ and Na_V_1.9^−/−^ animals by Student’s *t* test or 2-way ANOVA with Bonferroni post hoc test, as appropriate. In teased single-fibre flat sheet experiments, average spikes/s per stimulus were compared between genotype or before and after ring application of drugs/supernatants. All cytokine quantification data were analysed nonparametrically. If detectable levels of cytokines were present in control supernatants, Mann-Whitney *U* tests were performed, or else Wilcoxon signed rank tests comparing to a theoretical median of 0.0 were performed. Statistical significance was set at *P* < .05. Data are presented as mean ± SEM, *N* = number of animals, and *n* = number of observations.

### 2.11 Drugs

Stock concentrations of ATP (300 mM; water), PGE_2_ (1 mM; ethanol), bradykinin (10 mM; water), histamine (100 mM; water), 5HT (10 mM; water), atropine (10 mM; ethanol), indomethacin (3 mM; DMSO) and nifedipine (10 mM; DMSO) were all purchased from Sigma Aldrich (UK) and prepared as described. IS was prepared in advance and aliquots frozen until use. All compounds were diluted to working concentrations in buffer on the day of experimentation.

## 3 Results

### 3.1 Localization of Na_V_1.9 in visceral afferent neurons

**Fig. 1:**
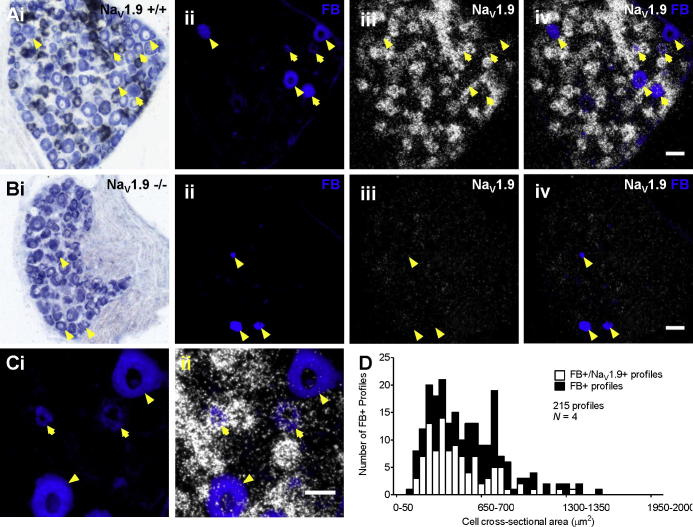
Expression of Na_V_1.9 mRNA in visceral colonic sensory neurons in mouse. Radiographic in situ hybridization of Na_V_1.9 mRNA transcript expression (bright field with Giemsa counterstain, i; polarized light, iii) in thoracolumbar dorsal root ganglia (DRG) section from Na_V_1.9^+/+^ (A) and Na_V_1.9^−/−^ (B) mice combined with retrograde labelling of sensory neurons from the colon with Fast Blue (FB) (FB only, ii; merge, iv; scale bar = 50 μm). (C) Expanded top-right region of Na_V_1.9^+/+^ ganglia section (A) showing 4 retrogradely labelled sensory neurons (scale bar = 30 μm). Yellow arrows indicate colon projecting neurons positive for Na_V_1.9 expression. Yellow arrowheads indicate colon projecting neurons negative for Na_V_1.9 expression. (D) Cross-sectional area histogram of Fast Blue–positive neurons from Na_V_1.9^+/+^ mice superimposed with those which also exhibit Na_V_1.9 mRNA transcript expression (*N* = 4).

We first examined the expression of Na_V_1.9 transcripts in colon-projecting thoracolumbar DRG sections of mouse via in situ hybridization (ISH). Under polarized light, Na_V_1.9-positive labelling was observed as clusters of silver grains visible over cells and was present in 69.0 ± 3.0% of all neurons comparable with previous studies in rat (Fig. 1) [11], [18]. Injections of FB retrograde tracer into the colon wall labelled 6.7 ± 1.8% of thoracolumbar DRG neurons in Na_V_1.9^+/+^ mice (Fig. 1Aii), and of these, 50.5 ± 3.3% expressed Na_V_1.9 transcripts. A similar proportion of FB-positive colonic neurons were found in Na_V_1.9^−/−^ mice (8.3 ± 0.9% vs Na_V_1.9^+/+^ FB positive, *P* = .46), indicating no loss or lesion of these cells. Importantly, no specific Na_V_1.9 expression was observed in sections from Na_V_1.9^−/−^ mice (Fig. 1B) and those incubated with excess unlabelled probe (data not shown). Size–frequency analysis of all neurons revealed that Na_V_1.9 mRNA was expressed to a higher extent in small (<20 μm diameter, 83.4 ± 2.9%) compared to medium to large (>20 μm diameter, 50.2 ± 2.9%) neurons. In the FB-positive population, a similar distribution was seen with 71.1 ± 10.9% small-diameter and 44.5 ± 3.6% medium-large diameter cells expressing Na_V_1.9 transcripts (Fig. 1D).

**Fig. 2:**
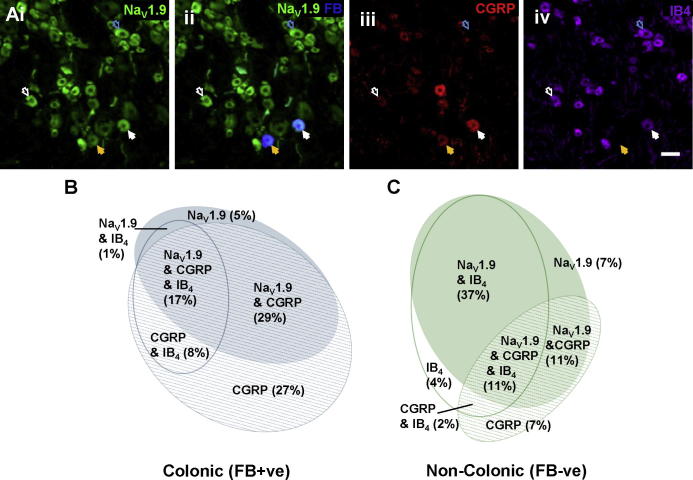
Expression of Na_V_1.9 in visceral colonic sensory neurons in rat. (A) Multiple fluorescent immunohistochemistry of Na_V_1.9 (i) and CGRP (iii) immunoreactivity and IB4 binding (iv; scale bar = 50 μm) in dorsal root ganglia from rat combined with retrograde labelling of sensory neurons from the colon (ii, merge of Na_V_1.9-IR and FB). Solid arrows indicate Fast Blue (FB)-positive neurons with Na_V_1.9, CGRP, and IB4 (white) or Na_V_1.9 and CGRP (orange). Open arrows indicate example FB-negative neurons with Na_V_1.9, CGRP, and IB4 (white) or Na_V_1.9 and IB4 (light blue). (B) Proportional Venn diagram of coimmunoreactivity for Na_V_1.9 and CGRP and IB4 binding in neuronal populations retrogradely labelled from the colon in rat. Approximately 13.3% of FB-positive neurons were negative for Na_V_1.9, CGRP, and IB4 binding (*N* = 3). (C) Proportional Venn diagram of coimmunoreactivity for Na_V_1.9 and CGRP and IB4 binding in noncolonic neuronal populations (FB negative) in rat. Approximately 21.6% of FB-negative neurons were negative for Na_V_1.9, CGRP, and IB4 binding (*N* = 3).

To further characterize Na_V_1.9 protein expression and confirm our mRNA distribution data, we used a commercially available Na_V_1.9 antibody in rat DRG sections. Additionally, this was performed in conjunction with CGRP and IB4 labelling, on the basis of the strong correlation between IB4 binding and Na_V_1.9 expression reported previously (Fang 2006). The Na_V_1.9 antibody produced nonspecific labelling in mouse DRG but proved highly specific in rat tissues [53]. No specific staining was seen in control experiments after omission of the Na_V_1.9 antibody or preincubation with blocking peptide (data not shown). Rat Na_V_1.9 immunoreactivity was observed in a similar proportion of total neurons (64.5 ± 2.4%, Fig. 2A) to mouse ISH, and colonic injection of FB labelled a similar proportion of neurones in rat DRGs (9.2 ± 1.6%). In agreement with the mouse ISH data in FB-positive neurones, 51.9 ± 5.8% of rat FB-positive cells stained positive for Na_V_1.9 protein. As expected, a greater proportion of CGRP-expressing cells (80.6 ± 8.3%) was observed in the colon-projecting population compared to all neurons (34.8 ± 1.1%, Fig. 2B, C), indicative of an enriched peptidergic population [42]. The extensive colocalization of Na_V_1.9 with IB4 staining predicted by previous studies was also observed in FB-negative neurons (73.4 ± 3.5% of Na_V_1.9; Table 2) [14]. By contrast, Na_V_1.9/IB4 colocalization was less extensive (34.7 ± 7.3% of Na_V_1.9) in FB-positive colonic populations, with an enrichment of colocalization with CGRP (from FB negative: 32.7 ± 1.1% to FB positive: 89.6 ± 7.8% of Na_V_1.9, Fig. 2B, C). As such, within the colon-projecting population, Na_V_1.9 appears to be almost exclusively expressed in CGRP-positive neurons.

**Table 2:** Comparison of Na_V_1.9 and CGRP immunohistochemical expression and IB4 binding in colonic and noncolonic sensory neurons.^a^

Characteristic	Colonic (FB positive; N = 4, *n* = 247)		Noncolonic (FB negative; N = 4, *n* = 2556)	
Na_V_1.9-IR	51.9 ± 5.8%		65.9 ± 3.0%	
CGRP-IR	80.6 ± 8.3%		30.0 ± 0.4%	
IB4 binding	26.0 ± 5.3%		54.1 ± 0.9%	
	% of Na_V_1.9	% of CGRP	% of Na_V_1.9	% of CGRP
Na_V_1.9 and CGRP-IR	89.6 ± 7.8%	57.7 ± 5.1%	32.7 ± 1.1%	71.7 ± 3.5%
	% of Na_V_1.9	% of IB4	% of Na_V_1.9	% of IB4
Na_V_1.9-IR and IB4 binding	34.7 ± 7.3%	68.5 ± 7.7%	73.4 ± 3.5%	89.2 ± 2.1%

FB, Fast Blue; CGRP, calcitonin gene–regulated peptide; IB4, isolectin-B4; IR, immunoreactivity.

a Percentage coexpression in terms of total stated marker.

### 3.2 Deletion of Na_V_1.9 inhibits colonic afferent responses to ATP and PGE_2_

**Fig. 3:**
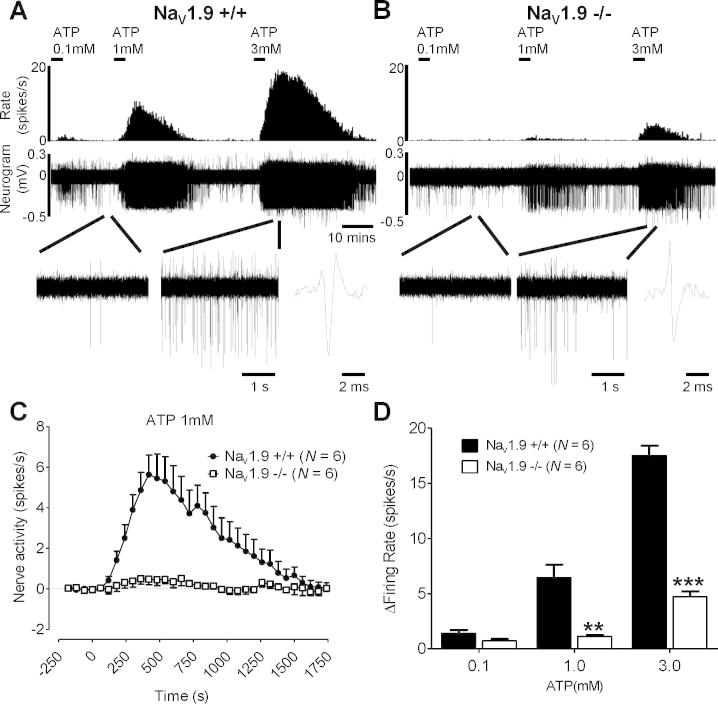
Effect of increasing concentrations of ATP on colonic splanchnic nerve activity in Na_V_1.9^+/+^ and Na_V_1.9^−/−^ mice. Example rate histogram and neurogram response in Na_V_1.9^+/+^ (A) and Na_V_1.9^−/−^ (B) mice to 0.1 mM, 1 mM, and 3 mM ATP. Below, expanded traces at baseline and after addition of 3 mM ATP and example action potential for each genotype. (C) Response profiles to addition of 1 mM ATP in Na_V_1.9^+/+^ (*N* = 6) and Na_V_1.9^−/−^ mice (*N* = 6). (D) Peak increase in nerve activity after addition of 0.1 mM, 1 mM, and 3 mM ATP above baseline activity in Na_V_1.9^+/+^ (*N* = 6) and Na_V_1.9^−/−^ (*N* = 6) mice. ^∗∗^*P* < .01, ^∗∗∗^*P* < .001.

**Fig. 4:**
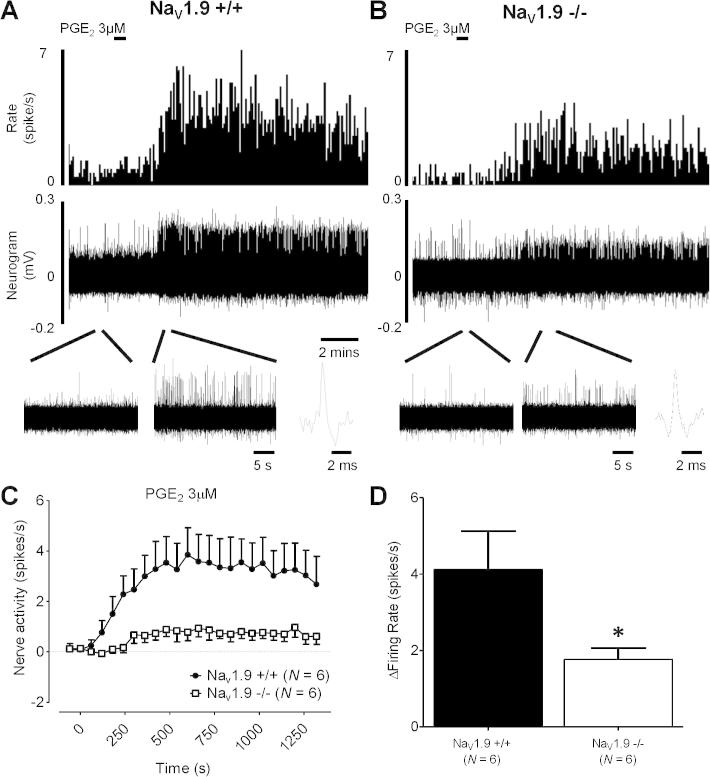
Effect of 3 μM PGE_2_ on colonic splanchnic nerve activity in Na_V_1.9^+/+^ and Na_V_1.9^−/−^ mice. Example rate histogram and neurogram response in Na_V_1.9^+/+^ (A) and Na_V_1.9^−/−^ (B) mice to 3 μM PGE_2_. Below, expanded traces at baseline and after addition of 3 μM PGE_2_ and example action potential for each genotype. (C) Response profiles to addition of 3 μM PGE_2_ in Na_V_1.9^+/+^ (*N* = 6) and Na_V_1.9^−/−^ mice (*N* = 6). (D) Peak increase in nerve activity after addition of 3 μM PGE_2_ in Na_V_1.9^+/+^ (*N* = 6) and Na_V_1.9^−/−^ (*N* = 6) mice. ^∗∗^*P* < .01, ^∗∗∗^*P* < .001.

To investigate the role of persistent tetrodotoxin-resistant sodium current activation in responses of visceral afferents to ATP and PGE_2_, we made extracellular recordings of the lumbar splanchnic nerve in Na_V_1.9^+/+^ and Na_V_1.9^−/−^ mice. Multiunit recordings provided an unbiased observation of the involvement of Na_V_1.9 in lumbar splanchnic nerve responses to superfused application of ATP and PGE_2_. Baseline firing was reduced in Na_V_1.9^−/−^ mice compared to control animals (0.7 ± 0.1 spikes/s, *N* = 41 vs 1.4 ± 0.3 spikes/s, *N* = 49; *P* < .05). The sequential application of increasing ATP concentrations led to robust and concentration-dependent increases in afferent firing (Fig. 3) in control mice. PGE_2_ (3 μm) also evoked comparable afferent excitation (Fig. 4). However, in Na_V_1.9^−/−^ mice, responses to ATP and to PGE_2_ were significantly diminished for the duration of the response. Even at the highest concentrations of ATP applied (3 mM), firing rates and nerve terminal sensitivity were reduced by 73% in Na_V_1.9^−/−^ afferents.

### 3.3 Effects of Na_V_1.9 deletion on responses of colonic afferents to IS and subsequent mechanical hypersensitivity

**Fig. 5:**
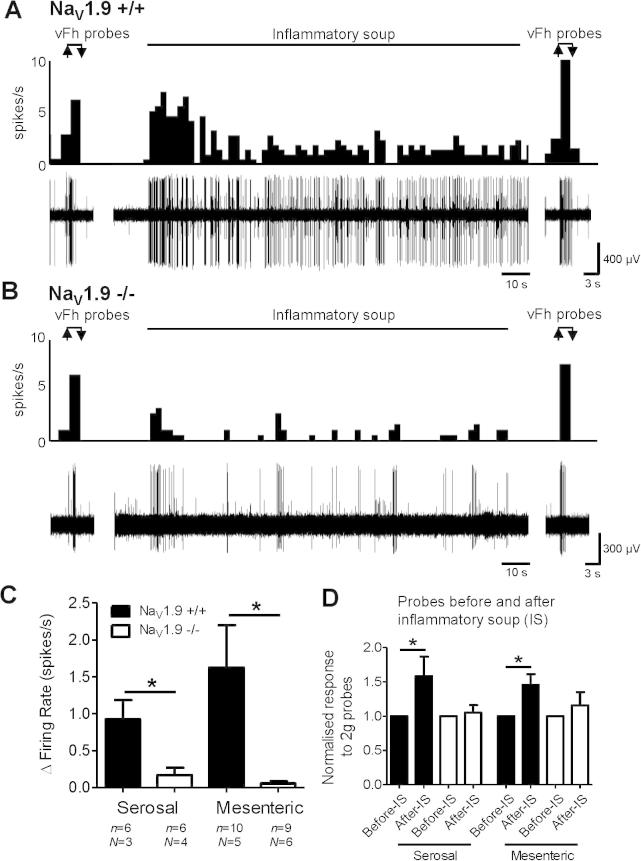
Chemical and mechanical stimulation of single-fibre mechanoreceptive fields in mouse colon. Example colonic nerve activity to ring application (2 min) of inflammatory soup (IS) over a receptive field located in the serosa of Na_V_1.9^+/+^ (A) and Na_V_1.9^−/−^ (B) mice. Probes with von Frey hair (2 g) were performed prior to and after IS application. (C) Response to IS ring application in serosal and mesenteric receptive fields. (D) Afferent hypersensitivity after IS application was observed in Na_V_1.9^+/+^ mice in both serosal and mesenteric units. This was not present in Na_V_1.9^−/−^ mice.

We next investigated a more generic role for Na_V_1.9 in afferent responses to multiple inflammatory mediators. To achieve this, single-fibre recordings were made in colonic flat sheet preparations, and receptive fields in the mesentery and serosa were identified. A mixture of multiple inflammatory mediators commonly used in pain studies was applied [15], [23], [49]. Discrete application of IS (bradykinin, ATP, histamine, serotonin, and PGE_2_) for 2 min in a small chamber surrounding the receptive field led to robust activation in 100% of serosal and 80% of mesenteric units in Na_V_1.9^+/+^ mice (Fig. 5). In line with our previous whole-nerve recordings of responses to ATP and PGE_2_, the increase in firing of single afferent fibres after IS was statistically reduced in Na_V_1.9^−/−^ mice, and only 43% of serosal (*P* < .05) and 56% of mesenteric (*P* < .05) units responded (Fig. 5C).

After responses to IS, mechanical hypersensitivity of colonic afferents was seen. We compared responses to 2 g vFh probing before and after IS application in control animals and observed a significant increase in responses after IS (*P* < .05, Fig. 5D). This is consistent with previous studies using the application of bradykinin [7]. IS-induced mechanical hypersensitivity to a 2 g probe was completely lost in Na_V_1.9^−/−^ mice (serosal, *P* = .76; mesenteric, *P* = .63), indicating that Na_V_1.9 may directly influence mechanical hypersensitivity of visceral afferents induced by IS.

### 3.4 Na_V_1.9 deletion reduces colonic afferent responses to human tissue–derived inflammatory supernatants

**Fig. 6:**
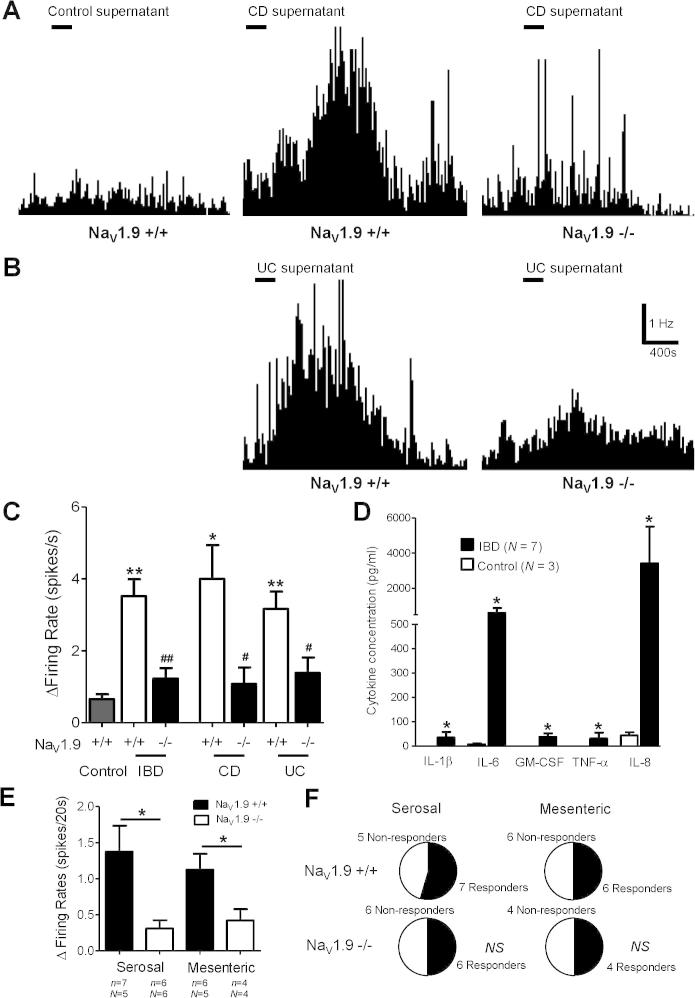
Effect of inflammatory bowel disease (IBD) supernatants on colonic nerve activity. (A) Example rate histograms of colonic nerve activity to control supernatant in Na_V_1.9^+/+^ mice and to Crohn disease (CD) tissue supernatant in Na_V_1.9^+/+^ and Na_V_1.9^−/−^ mice. (B) Example rate histograms of colonic nerve activity to ulcerative colitis (UC) supernatant in both Na_V_1.9^+/+^ and Na_V_1.9^−/−^ mice. (C) Peak change in activity after addition of control (*N* = 3), IBD supernatants in Na_V_1.9^+/+^ (*N* = 7), and Na_V_1.9^−/−^ mice (*N* = 6). In addition, CD and UC responses are plotted separately (right; *N* = 3–4 per genotype). ^∗^*P* < .05, ^∗∗^*P* < .01 vs control; ^#^*P* < .05, ^##^*P* < .01 Na_V_1.9^+/+^ vs Na_V_1.9^−/−^. (D) Cytokine (IL-1β, IL-6, GM-CSF, TNF-α, and IL-8) levels in IBD (*N* = 7) and control (*N* = 3) supernatants applied to afferent nerve recordings. ^∗^*P* < .05 vs control. (E) Responses to ring application of CD supernatants over serosal and mesenteric receptive fields in Na_V_1.9^+/+^ and Na_V_1.9^−/−^ mice. (F) Number of responsive vs nonresponsive serosal and mesenteric units when CD supernatants were applied.

We investigated whether ablation of Na_V_1.9 would also alter responses to supernatants derived from inflamed human tissue. To do this, resected bowel tissue from patients undergoing surgery for inflammatory bowel disease (IBD; CD and UC) was incubated in Krebs solution to generate individual IBD supernatants. Separately, macroscopically normal tissue taken from bowel cancer resections (>10 cm away from the cancer itself, cancer margins, and lymph nodes) was used to generate control supernatants. These control supernatants enabled possible excitatory effects caused by surgical manipulation, removal of tissue, and temporal ischaemia to be discounted from afferent activation driven by inflammatory mediators released from native tissue. Importantly, no further steps were taken to concentrate the tissue supernatant in any way. To confirm the inflammatory status of the supernatants, cytokine quantification for IL-6, IL-8, IL-1β, GM-CSF, and TNF-α was performed. In control supernatants, there were detectable levels of IL-6 (7.5 ± 3.3 pg/mL) and IL-8 (43.4 ± 13.8 pg/mL), with levels of the remaining cytokines below detection limits (Fig. 6D). Cytokine levels were all significantly elevated in IBD supernatants (IL-1β: 35.4 ± 22.7; IL-6: 598.6 ± 276.3; GM-CSF: 37.5 ± 14.9; TNF-α: 31.5 ± 23.6; IL-8: 3419.3 ± 2083.7 pg/mL; each cytokine vs control, *P* < .05), with cytokine levels greatest in supernatants generated from CD tissue compared to those of UC [(IL-1β: 8.8 ± 3.6; IL-6: 186.4 ± 32.5; GM-CSF: 15.7 ± 3.0; TNF-α: 6.2 ± 2.1; IL-8: 1040.0 ± 231.2 pg/mL) CD: (IL-1β: 75.7 ± 46.3; IL-6: 1157.0 ± 507.8; GM-CSF: 66.8 ± 27.9; TNF-α: 64.0 ± 54.4; IL-8: 6446.0 ± 4715.0 pg/mL)]. It should be mentioned that the influence on cytokine production by immunosuppressant and anti-TNF-α antibody therapies was not accounted for during patient selection. Supernatants were applied to mouse whole-nerve lumbar splanchnic recordings in a cannulated tubular preparation, similar to that used for ATP and PGE_2_ application. IBD supernatants elicited a robust increase in afferent firing compared to control supernatants in wild-type animals (3.5 ± 0.5 spikes/s vs control supernatant 0.7 ± 0.1 spikes/s, *P* < .01, Fig. 6C). In Na_V_1.9^−/−^ mice, the response to IBD supernatants was greatly attenuated and almost comparable in magnitude to control supernatant response in wild-type mice (1.2 ± 0.3 spikes/s, vs Na_V_1.9^+/+^ mice, *P* < .01). We then investigated the effect of application of CD supernatants to individual isolated receptive fields using single-fibre recordings in flat sheet preparations. Responses to application of CD supernatant were seen in 64% of serosal (1.37 ± 0.36 spikes/20 s) and 50% of mesenteric afferents (1.13 ± 0.22 spikes/20 s) (Fig. 6E). The proportions of units responding in Na_V_1.9^−/−^ mice were comparable (*P* = 1.00, Fig. 6F); however, the degree of afferent activation elicited by application of CD supernatant to receptive fields was significantly less (serosal: 0.31 ± 0.11 spikes/20 s, *P* < .05 vs Na_V_1.9^+/+^; mesenteric: 0.42 ± 0.16 spikes/20 s, *P* < .05 vs Na_V_1.9^+/+^). Unlike effects of experimental IS, no mechanical hypersensitivity was observed after CD supernatant in either Na_V_1.9^+/+^ or Na_V_1.9^−/−^ mice to 2 g vFh probe (data not shown), regardless of whether afferents showed direct excitation to supernatant. However, it is clear that Na_V_1.9 contributes to direct afferent excitation by both artificial and natural inflammatory milieu of differing composition.

### 3.5 Deletion of Na_V_1.9 increases mechanosensory activation thresholds of afferent fibres and reduces maintenance of repeated responses

**Fig. 7:**
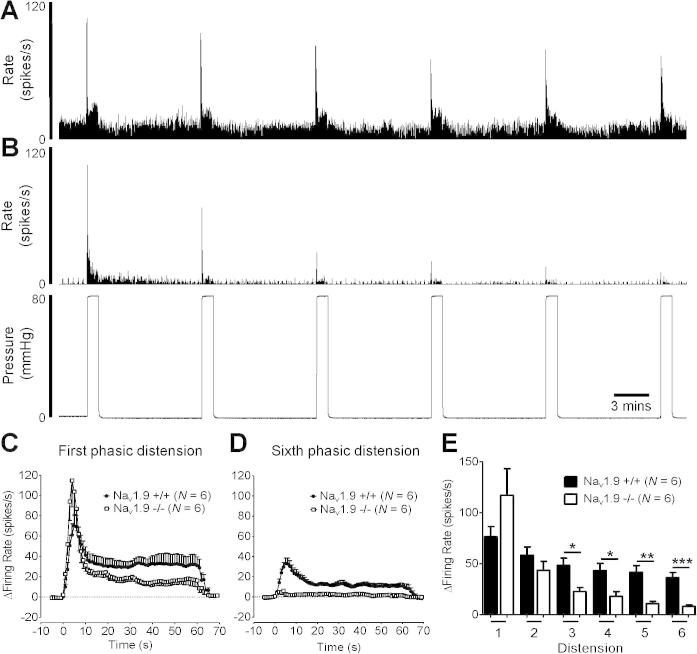
Example rate histogram of colonic splanchnic nerve response to repeat phasic distension (0–80 mm Hg; 60 s; 10 min intervals) in Na_V_1.9^+/+^ (A) and Na_V_1.9^−/−^ mice (B). Average response profiles to the first (C) and sixth (D) phasic ramp distension in Na_V_1.9^+/+^ and Na_V_1.9^−/−^ mice. (E) Peak change in firing rate during sequential phasic distensions (^∗^*P* < .05; ^∗∗^*P* < .01; ^∗∗∗^*P* < .001, unpaired *t* test).

Our demonstration of a role for Na_V_1.9 in mechanical hypersensitivity in response to IS prompted us to also determine how Na_V_1.9 influences mechanosensation. To do this, we used whole-nerve recordings of distension responses in a cannulated tubular preparation of the distal colon, plus single-fibre recording techniques in a flat sheet preparation. Phasic distension of the bowel to 80 mm Hg intraluminal pressure led to a robust initial increase in afferent activity after adapting to a plateau phase for the remainder of the 1 min distension (Fig. 7). Second and third repeat distensions (at 9 min intervals) evoked smaller peak responses until a stabilized peak response was reached by the fourth to sixth distension. In Na_V_1.9^−/−^ mice, peak responses to first distension were similar to control animals (*P* = .17, Fig. 7C). However, afferent responses to subsequent repeat distension in Na_V_1.9^−/−^ mice showed greater tachyphylaxis with significant reductions observed by the third to sixth distensions (*P* < .001, Fig. 7B, E).

**Fig. 8:**
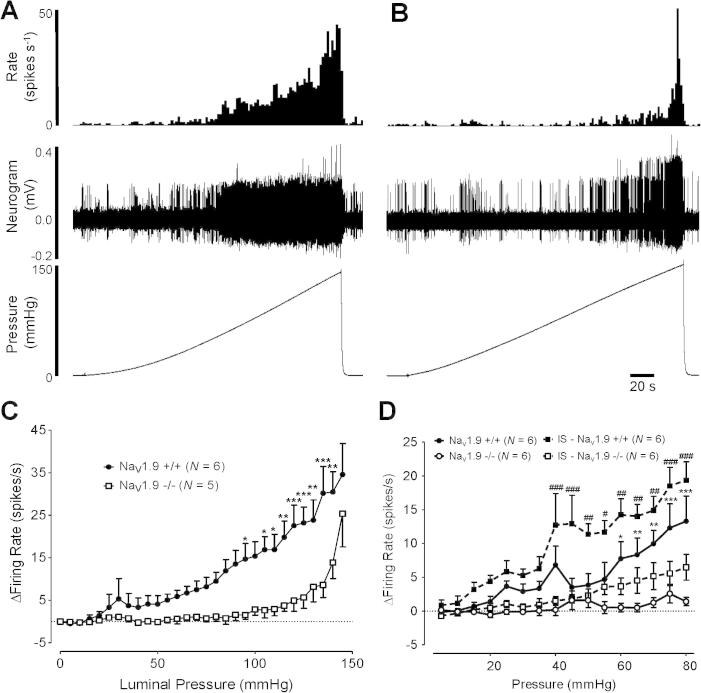
Example rate histogram and raw trace of colonic splanchnic nerve response to ramp distension (0–145 mm Hg) in Na_V_1.9^+/+^ (A) and Na_V_1.9^−/−^ (B) mice with intraluminal pressure trace. (C) Firing rates to distension pressures of <95 mm Hg were greatly attenuated in Na_V_1.9^−/−^ animals compared to Na_V_1.9^+/+^ mice (*N* = 6), with an increase in firing at greater pressures in Na_V_1.9^−/−^ mice (*P* < .0001, 2-way ANOVA with Bonferroni’s post hoc test, ^∗^*P* < .05; ^∗∗^*P* < .01; ^∗∗∗^*P* < .001). (D) Firing rates to noxious 80 mm Hg ramp distension in Na_V_1.9^+/+^ and Na_V_1.9^−/−^ mice were increased by intraluminal perfusion with inflammatory soup (IS) (*P* < .0001, Na_V_1.9^+/+^ vs Na_V_1.9^−/−^, 2-way ANOVA with Bonferroni’s post hoc test, ^∗^*P* < .05; ^∗∗^*P* < .01; ^∗∗∗^*P* < .001; *P* < .0001, Na_V_1.9^+/+^ in presence of IS vs Na_V_1.9^−/−^ in presence of IS, 2-way ANOVA with Bonferroni’s post hoc test, ^#^*P* < .05, ^##^*P* < .01, ^###^*P* < .001).

These findings suggested a particular role for Na_V_1.9 in responses to persistent stimuli. To investigate this role further, we used a slow ramp distension paradigm. Initial ramp distensions up to 80 mm Hg intraluminal pressure evoked maintained whole-nerve responses with a linear correlate between afferent activity and pressure, reaching a maximum firing rate of 13.3 ± 3.7 spikes/s (*N* = 6) in control animals. Na_V_1.9^−/−^ mice, by contrast, elicited minimal activity during comparable ramp distensions, with a small burst of firing observed at ∼30 mm Hg (maximum 1.4 ± 0.6 spikes/s, *N* = 6, *P* < .0001). We reasoned that rapid phasic distension in experiments described above develops a more substantial stimulus to mechanoreceptive afferents compared to a slow ramp fill, even up to equivalent final pressures (80 mm Hg). Therefore, it should be possible to overcome the lack of response to slow ramp distension by increasing the stimulus to supraphysiologic distension pressures. The maximum distension pressure achievable in mouse colon before rupture was determined at 159.6 ± 4.3 mm Hg (*N* = 10). Ramp distension to 145 mm Hg was thus used to provide a stronger stimulus. Ramp distensions beyond 80 mm Hg continued to linearly increase afferent firing rates to a maximum of 34.6 ± 7.3 spikes/s in Na_V_1.9^+/+^ mice (Fig. 8A). However, in Na_V_1.9^−/−^ mice, afferent firing remained just above baseline (<2 spikes/s) up to ∼95 mm Hg, above which an exponential increase in firing was observed reaching firing rates almost comparable to Na_V_1.9^+/+^ animals at 145 mm Hg (25.4 ± 7.8 spikes/s, Fig. 8B, C). Mechanical sensitization of responses to noxious ramp distensions (80 mm Hg) was investigated by intraluminal perfusion of IS. In Na_V_1.9^+/+^ mice, perfusion of IS led to direct activation of basal afferent firing rates, which was not observed in Na_V_1.9^−/−^ mice (3.8 ± 0.6 spikes/s, *N* = 5 vs 0.0 ± 0.4 spikes/s *N* = 6; *P* < .001, unpaired *t* test). Responses to ramp distension during IS perfusion were potentiated in Na_V_1.9^+/+^ mice, an effect not observed in Na_V_1.9^−/−^ mice up to 50 mm Hg (Fig. 8D). At greater ramp distension pressures, sensitized responses were observed in Na_V_1.9^−/−^ tissues compared to control experiments, which may reflect either the sensitization of afferent fibres negative for Na_V_1.9 or recruitment of alternative sensitizing mechanisms within Na_V_1.9-containing fibers.

**Fig. 9:**
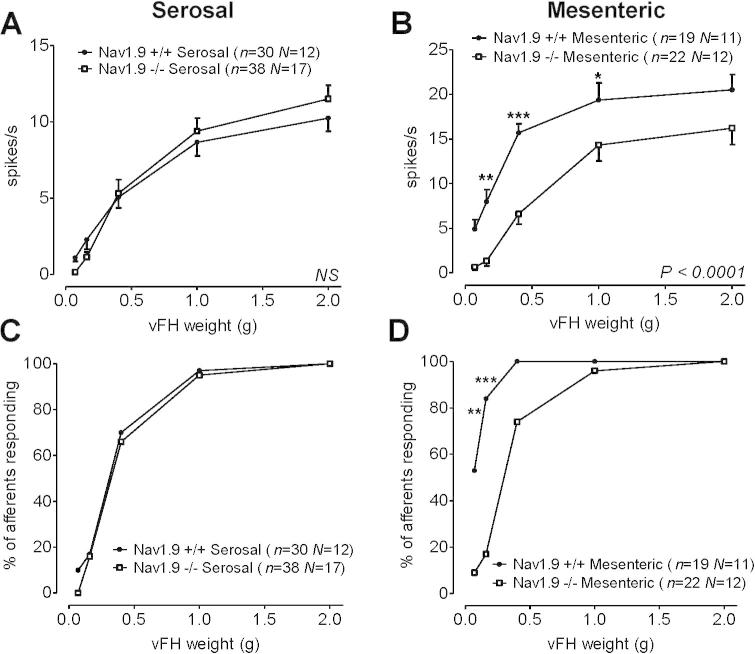
Stimulus–response curves to von Frey hair (vFh) probing for afferent fibre subtypes in Na_V_1.9^+/+^ and Na_V_1.9^−/−^ mice. Stimulus–response curves to vFh probing (0.07–2 g) for serosal (A) and mesenteric (B) afferent fibres (2-way ANOVA with Bonferroni’s post hoc test, ^∗^*P* < .05; ^∗∗^*P* < .01; ^∗∗∗^*P* < .001). Associated activation thresholds of vFh probing for serosal (C) and mesenteric (D) afferent fibres (Fisher’s exact test at each probe weight, ^∗∗^*P* < .01; ^∗∗∗^*P* < .001).

The deficits in afferent mechanosensation that we observed could be due to increased adaptation above threshold and/or altered thresholds for activation in particular fibers. To identify which distension-sensitive afferent subtype was altered in Na_V_1.9^−/−^ mice, we used established single-fibre recording techniques in a colonic flat sheet preparation. Although von Frey probes enable the highly reproducible and directed stimuli required to interrogate specific afferent subtypes, they are unlikely to represent an in vivo stimulus encountered by the intact bowel. There are 4 subtypes of lumbar splanchnic nerve afferents characterized by their location and responses to stroke, von Frey probe, and circumferential stretch: mucosal, muscular, mesenteric, and serosal afferents [6]. Stimulus–response curves to probe with increasing weight of vFhs of serosal and muscular units was unchanged between genotype (*P* = .94 and *P* = .24, respectively), with no significant differences observed in either mechanosensory thresholds or responses to circumferential stretch of the preparation (Fig. 9). We did observe a significant reduction in mesenteric afferent responses to vFh probing, specifically lower-intensity probing (0.16–1 g vFh), with a corresponding increase in mechanosensory threshold at these intensities (Fig. 9B, E). No difference in conduction velocity was observed between mechanoreceptors in control and Na_V_1.9^−/−^ mice (mean conduction velocity Na_V_1.9^+/+^ mice, 0.50 ± 0.03 m/s, *n* = 33, *N* = 10 vs Na_V_1.9^−/−^ mice, 0.52 ± 0.05 m/s, *n* = 52, *N* = 16; *P* = .82), signifying that action potential propagation is unaffected by loss of Na_V_1.9. Together, these data suggest that high-intensity mechanical stimulation of colonic afferents (eg, 2 g vFh probing, >95 mm Hg ramp distension, and initial rapid phasic distension) is unaffected by Na_V_1.9 gene deletion. However, knockout of Na_V_1.9 appears to decrease the sensitivity of mesenteric afferents to low-intensity stimulation, which accounts for the significant deficits observed in whole-nerve recordings during ramp distension.

## 4 Discussion

Our data demonstrate that Na_V_1.9 is required for direct excitatory responses of colonic afferents to noxious inflammatory mediators and is the subsequent development of mechanical hypersensitivity. We show that Na_V_1.9 is involved in determining the mechanosensory thresholds of a subset of colonic afferents with receptive fields in the mesenteric attachments, and we provide evidence of a significant role for Na_V_1.9 during persistent (repeat phasic distensions) or sustained (ramp distension) noxious distension of the colon. Finally, we show that Na_V_1.9 is important in the activation of visceral nociceptors located in the colonic serosa and mesentery by mediators released during human inflammatory disease.

Given the polymodal nature of visceral afferents responsive to noxious stimuli, there are likely points of convergence in the molecular signalling pathways underlying action potential generation at their endings. These may provide valuable targets by which future treatments could counteract mechanical hypersensitivity induced by on-going inflammation. Voltage-gated sodium channels may represent such a convergence point. Of the peripherally expressed sodium channels, valuable insight has been obtained regarding the function of Na_V_1.7 and Na_V_1.8 in visceral chronic pain, not least due to causal links to human pain syndromes [17], [40]. However, functional evidence for a role of Na_V_1.9 in the activation of visceral afferents has not been extensively explored. The data presented here suggest that Na_V_1.9 represents an important regulator of specific aspects of sustained visceral afferent excitability, especially in responses to inflammatory mediators.

It is known that the persistent sodium current defined by Na_V_1.9 can be greatly enhanced by inflammatory mediators acting at GPCRs via G_q/11_/PKC and G_i/o_/PKA intracellular signalling pathways [2], [10], [30], [44]. In some studies, this has required the application of multiple mediators [30], whereas in other studies, the application of single mediators alone is sufficient to increase Na_V_1.9 currents [2], [44]. Our study extends these observations by examining the contribution of Na_V_1.9 to nociceptor activation at the level of the nerve ending. We observe significantly reduced colonic afferent firing in Na_V_1.9^−/−^ mice to individually applied ATP and PGE_2_, even at supramaximal concentrations, demonstrating that Na_V_1.9 is required in the activation of nerve fibres by these mediators. These findings suggest that the application of single mediators is sufficient to enhance Na_V_1.9 currents and trigger action potentials in visceral afferents. However, it is important to stress that other inflammatory mediators may also be present at the afferent terminal due to ongoing interactions with immune cells or gut microbiota. As a consequence, we cannot rule out the possibility that multiple mediators may be acting to enhance Na_V_1.9 currents. Another consideration is that both ATP and PGE_2_ can enhance currents generated by other sodium channels (eg, Na_V_1.8 [2]) or ion channels [45], [50]; however, our data suggest that visceral afferent excitability to these mediators appears highly dependent on Na_V_1.9. The magnitude of the effect to ATP was potentially surprising given the relatively small proportion of colon afferent somata responsive to P2X_1,3_ agonist (30–35% [25]) and may reflect the engagement of alternative purinoceptors, including P2Y receptors.

In addition to attenuating responses to single mediators, in Na_V_1.9^−/−^ mice we also observed a substantial reduction in serosal and mesenteric afferent firing after the application of an experimental IS (consisting of bradykinin, histamine, PGE_2_, ATP, and 5HT). This indicates that Na_V_1.9 is required for visceral afferent activation to a range of inflammatory mediators. To explore this further, we generated supernatants by incubating resected tissue from patients with IBD. These disease derived IBD supernatant–evoked increases in afferent activity in whole-nerve recordings from mouse colon, and in single-unit recordings from serosal and mesenteric afferents; both were significantly attenuated in Na_V_1.9^−/−^ mice. Collectively, this suggests that during human inflammatory disease where multiple inflammatory stimuli are present, Na_V_1.9 contributes significantly to visceral afferent activation.

Deletion of Na_V_1.9 also caused significant deficits in mechanosensation and in the development of mechanical hypersensitivity after application of inflammatory mediators. These deficits were investigated using different mechanical stimuli, including von Frey probing of isolated visceral nociceptor receptive fields, repeated phasic distension of the colon at noxious pressures, and ramp distension of the colon into the noxious pressure range. Such studies have proven invaluable in the development of our current understanding of how noxious mechanical stimulation of the gut is transduced by visceral nociceptors [8], [16], [38]. We used vFh probes to elicit brief, intense, and graded mechanical stimuli. These responses were sensitized in both serosal and mesenteric afferents after application of IS, an effect that is absent in Na_V_1.9^−/−^ mice. Furthermore, the response to noxious ramp distension was also sensitized by IS, which, at least during lower distension pressures, was dependent on Na_V_1.9. This is consistent with the proposed role for Na_V_1.9 in regulating afferent nerve terminal sensitization during inflammation [31].

We observed reduced baseline mechanosensitivity in mesenteric but not serosal or muscular afferents, suggesting that Na_V_1.9 may contribute to the regulation of excitability in this afferent subtype. Interestingly, mechanosensitivity is reduced in serosal, but not mesenteric, afferents in ASIC2^−/−^ mice [38], [48]. It remains to be seen whether differences in the expression of mechanotransducers or other known regulators of neuronal excitability (such as T-type calcium channels or HCN2) present in serosal vs mesenteric afferents contributes to the importance of Na_V_1.9 in regulating afferent firing remains [12], [46].

Next we showed that the activation of visceral afferents by an initial rapid phasic distension is unaffected by Na_V_1.9 deletion. This observation supports the data generated using high-intensity vFh (2 g) stimulation which produced comparable responses across all types of units regardless of genotype. It suggests that the reduction in afferent excitability caused the loss of Na_V_1.9 may be overcome by greater mechanical stimulation, and presumably greater activation of stimulus-transducing channels. In contrast to the initial phasic distension, Na_V_1.9 is required for the maintenance of afferent sensitivity to persistent mechanical stimuli. The desensitization in responses typically seen to repeat colorectal distension [22], [47] did not stabilize in Na_V_1.9^−/−^ mice but instead continued to decline with subsequent distensions. One explanation for this observation that is worthy of further investigation is that repeated mechanical stimuli may progressively desensitise activity in mechanosensitive channels, such as TRPV4, TRPA1, and ASIC3; however, a concurrent up-regulation of Na_V_1.9 maintains a consistent level of afferent activation. This mechanism could be crucial in vivo, where pain is evoked by repeated contractions of the colon around a bolus or stricture [9]. Consistent with this proposed role for Na_V_1.9 in persistent or sustained stimuli, we observed substantial reductions in the afferent response to ramp colorectal distension in Na_V_1.9^−/−^ tissue at all pressures, including those within the noxious range. When these pressures were extended into the supramaximal range (100–150 mm Hg), increases in afferent activity were observed in both genotypes. This is in keeping with our previous suggestion that increased stimulus strength may overcome the loss of excitability after deletion of Na_V_1.9.

**Fig. 10:**
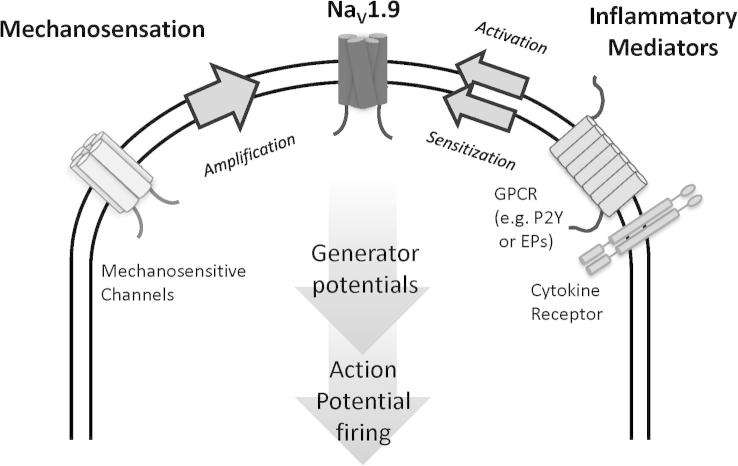
Schematic of Na_V_1.9 interacting with membrane-bound receptors and ion channels in a visceral afferent terminal. When present at the visceral afferent terminal, Na_V_1.9, as well as contributing to setting the resting membrane potential, acts to amplify generator potentials evoked by mechanosensitive channels and functions as a key transducer of inflammatory mediators and other sensitizing stimuli.

Considerable efforts were taken to ensure that changes observed in this study were not confounded by the loss of Na_V_1.9 within intrinsic primary afferents neurones (IPANs) of the enteric nervous system (ENS) [37], [43]. Potential impact of the loss of Na_V_1.9 in IPANs on local motor reflexes was controlled by inhibiting smooth muscle contractility through the addition of nifedipine and atropine to the perfusion buffer. To reduce the probability of recording activity from intestinofugal neurones within the ENS, our recordings were made distil to the primary site of intestinofugal afferent termination in the inferior mesenteric ganglia [29]. We believe these steps, in conjunction with the magnitude of the effects observed and paucity of evidence for cross-talk between the ENS and extrinsic afferents, strongly suggest that the changes we observed are due to modulation of neuronal excitability by Na_V_1.9 in visceral extrinsic afferents [33]. Visceral afferents responsive to noxious stimuli are relatively unspecialized anatomically and therefore require mechanisms for regulating neuronal excitation induced by a range of innocuous and noxious mechanical and inflammatory stimuli [48]. Indeed, visceral nociceptors, in particular serosal or mesenteric afferents, are prone to modulation by inflammatory mediators, and they may be recruited after being previously silent [5], [7], [15], [16]. Our results suggest that Na_V_1.9 represents a significant mechanism responsible for regulating visceral nociceptor sensitivity to sustained inflammatory and mechanical stimuli (Fig. 10). The recent identification of gain-of-function *SCN11A* mutations in humans resulting in both familial episodic pain and painful neuropathy provides important evidence indicating that Na_V_1.9 is present in pain-sensing nerves in humans and that modulating Na_V_1.9 function produces pain [20], [54].

In summary, our data support an important physiological role for Na_V_1.9 in the regulation of visceral afferent sensitivity during inflammation, and one that will likely have clinical relevance to human visceral pain. Further studies are required to fully understand how inflammatory pathways interact with Na_V_1.9 within the afferent fibre. The role of distinct sodium channel activity in human afferent function has been indirectly addressed here by combining human ex vivo samples with the use of null mutant mice. This approach allows us to conclude that Na_V_1.9 is an important target for the treatment of visceral pain.

## Conflict of interest statement

WW is an employee of Neusentis (Pfizer Ltd). The other authors report no conflict of interest.
